# Weight of Clinical and Social Determinants of Metabolic Syndrome in People Living with HIV

**DOI:** 10.3390/v14061339

**Published:** 2022-06-20

**Authors:** Maria Mazzitelli, Paolo Fusco, Michele Brogna, Alfredo Vallone, Laura D’Argenio, Giuseppina Beradelli, Giuseppe Foti, Carmelo Mangano, Maria Stella Carpentieri, Lucio Cosco, Paolo Scerbo, Armando Priamo, Nicola Serrao, Antonio Mastroianni, Chiara Costa, Maria Teresa Tassone, Vincenzo Scaglione, Francesca Serapide, Enrico Maria Trecarichi, Carlo Torti

**Affiliations:** 1 Unit of Infectious and Tropical Diseases, Department of Medical and Surgical Sciences, “Magna Graecia” University, 88100 Catanzaro, Italy; paolofusco89@gmail.com (P.F.); costach65@gmail.com (C.C.); mariateresatassone90@gmail.com (M.T.T.); vincenzo.scaglione91@gmail.com (V.S.); francescaserapide@gmail.com (F.S.); em.trecarichi@unicz.it (E.M.T.); torti@unicz.it (C.T.); 2 Infectious and Tropical Diseases Unit, Padua University Hospital, 35128 Padua, Italy; 3 Jazzolino Hospital, 89900 Vibo Valentia, Italy; brognam@libero.it (M.B.); alfredo.vallone@aspvv.it (A.V.); laura.dargenio@aspvv.it (L.D.); 4 Unit of Infectious and Tropical Diseases, “Giovanni Paolo II” Hospital, 88046 Lamezia Terme, Italy; giuseppina.berardelli@asp.cz.it; 5 Unit of Infectious and Tropical Diseases, “Bianchi-Melacrino-Morelli” Hospital, 89121 Reggio Calabria, Italy; fotigiuseppe@tin.it (G.F.); mangano.carmelo@alice.it (C.M.); carpentieri.mariastella@gmail.com (M.S.C.); 6 Unit of Infectious and Tropical Diseases, “Pugliese-Ciaccio” Hospital, 88100 Catanzaro, Italy; lucio.cosco@alice.it (L.C.); scr2002@hotmail.com (P.S.); armando1956@alice.it (A.P.); 7 Unit of Infectious and Tropical Diseases, “San Giovanni di Dio” Hospital, 88900 Crotone, Italy; n.serrao@libero.it; 8 Unit of Infectious and Tropical Diseases, “Annunziata” Hospital, 87100 Cosenza, Italy; antoniomastroianni@yahoo.it

**Keywords:** HIV, PLWH, metabolic syndrome, non-communicable diseases, diabetes, dyslipidemia, AIDS

## Abstract

**Background.** Comorbidities in people living with HIV (PLWH) represent a major clinical challenge today, and metabolic syndrome (MTBS) is one of the most important. **Objective.** Our objective was to assess the prevalence of MTBS and the role of both clinical/socio-behavioral risk factors for MTBS in a cohort of PLWH. **Methods.** All PLWH, over 18 years of age, attending all Infectious Disease Units in Calabria Region (Southern Italy) for their routine checks from October 2019–January 2020 were enrolled. MTBS was defined by NCEP-ATP III criteria. Logistic regression analysis was performed to assess factors significantly associated with the main outcome (MTBS). **Results.** We enrolled 356 PLWH, mostly males (68.5%), with a mean age of 49 years (standard deviation: 12), including 98 subjects with and 258 without MTBS. At logistic regression analysis, a statistically significant association was found between MTBS and alcohol use, osteoporosis, polypharmacy, and a history of AIDS. **Conclusions.** Identifying and addressing risk factors, including those that are socio-behavioral or lifestyle-related, is crucial to prevent and treat MTBS. Our results suggest the importance of implementing educational/multidimensional interventions to prevent MTBS in PLWH, especially for those with particular risk factors (alcohol abuse, osteoporosis, previous AIDS events, and polypharmacy). Moreover, alcohol consumption or abuse should be routinely investigated in clinical practice.

## 1. Introduction

Due to increased life expectancy and efficacy of newer antiretrovirals, the burden of non-infectious comorbidities in people living with HIV (PLWH) is increasing [1,2]. Indeed, cardiovascular disease, metabolic complications, cancer, and bone disorders are the most frequent comorbidities in this population [3,4]. Among these, metabolic syndrome (MTBS) is one of the most frequent [5]. Therefore, HIV became a chronic disease for which management of non-communicable diseases (NCDs) remains to date the major clinical challenge [6]. One of the most important issues is the management of the metabolic disease because MTBS is not only the main drivers of major cardiovascular events, but it is also associated with an increased risk of respiratory disorders and malignancies [7,8] and possible side effects due to polypharmacy [9]. This is the reason why dedicated clinics and services for a multidimensional approach to ageing PLWH have been implemented over time [10].

Data about prevalence of the metabolic syndrome in people with HIV are not definitive. Indeed, some data reported the prevalence of MTBS to be about 30%, comparable with the prevalence of MTBS in the general population, while other studies reported that prevalence was slightly higher in PLWH than in the general population [11,12]. Beyond the above-mentioned criteria, social factors and lifestyle have been identified as contributors to the risk of MTBS, and control of some social habits was also associated with prevention of MTBS [11]. Moreover, recently it has been demonstrated that socioeconomic and lifestyle differences between people with and without HIV could lead to a 2.5-fold increased life-year loss [13], and for PLWH, specific factors such as chronic inflammation and type of antiretroviral therapy could contribute to increases risk of metabolic alterations leading to other chronic diseases [14,15].

In this study, we aimed at assessing prevalence of MTBS in PLWH in southern Italy and both clinical and social determinants associated with its presence.

## 2. Materials and Methods

This observational study was coordinated by the Infectious and Tropical Diseases Unit of “*Mater Domini*” teaching hospital in Catanzaro (Italy) and was conducted in accordance with the Declaration of Helsinki and the principles of Good Clinical Practice [16]. The local ethical committee (Calabria Region) approved the study protocol on 19 July 2018. Written informed consent was obtained from all subjects enrolled. Participation in the survey was proposed to all PLWH older than 18 years, attending the Infectious and Tropical Diseases Units (ITDUs) in Calabria (cities of Catanzaro—two centers—, Cosenza, Crotone, Lamezia Terme, Reggio Calabria, and Vibo Valentia) for their routine clinical checks from 1 October 2019 to 31 January 2020. Pregnant women and people aged under 18 years were excluded. The study population was divided into two groups: PLWH with MTBS and PLWH without MTBS. According to NCEP-ATP III criteria, metabolic syndrome (MTBS) was defined by the presence of three or more of the following parameters: waist circumference greater than 102 cm in males and 88 in females, blood pressure higher than 135/80 mmHg, fasting blood glucose greater than 100 mg/dL, HDL lower than 50 mg/dL for men and 40 mg for women, and triglycerides level higher than 150 mg/dL [17].

Data regarding demographics (age, gender, country of origin), clinical history, HIV-related characteristics (viral load, CD4 + T cell count, AIDS-defining illnesses in the past medical history) and all comorbidities, co-medications, risk factors and lifestyle-related characteristics (smoking habit, alcohol consumption, physical exercise), and blood test results were collected. Data on the level of education were collected, setting up a highest level of education up to 16 years (primary school, 5 years; secondary school, 3 years; high school, 5 years; university, 3 or more years). Data on comorbidities were retrieved by clinical health records. Hypertension was defined by its presence in the medical history or by anti-hypertensive agents among comedication. Physical activity was assessed by using WHO definitions according to age [18]. Chronic kidney disease was considered if mentioned in the medical history or in subject with an estimated glomerular filtration rate below 90 mL/min [19]. Excessive alcohol intake was measured by using definitions of Italian Ministry of Health: intake of 2 or more or 1 or more alcoholic units/day (1 units = 12 g of alcohol) for men and women, respectively, or experiencing episodes of binge drinking (intake of 5 or more or 4 or more alcoholic units at once for men and women, respectively) [20]. Weight and height to calculate body mass index (BMI) and waist circumferences (to establish MTBS criteria) were measured during clinical check. Polypharmacy was defined as the intake of 5 or more medications in the same patient [21]. Each participant was given a unique study identification number, and data regarding each patient were transferred onto an Excel database.

Continuous variables were compared by Student’s *t*-test for normally distributed variables and the Mann–Whitney U test for non-normally distributed variables. Categorical variables were evaluated using the χ^2^ or two-tailed Fisher’s exact test. Odds ratios (ORs) and 95% confidence intervals (CIs) were calculated to evaluate the strength of any association that emerged. Values are expressed as mean (± standard deviation) (continuous variables) or as percentages of the group from which they were derived (categorical variables). Two-tailed tests were used to determine statistical significance; a *p*-value of <0.05 was significant. Multivariate analysis was used to explore any possible correlation with the main outcome (MTBS). For this analysis, we used logistic regression and incorporated variables found to be significant in univariate testing. All statistical analyses were performed using the Intercooled Stata program, version 11, for Windows (Stata Corporation, College Station, TX, USA).

## 3. Results

Over the study period, we enrolled 356 PLWH, namely 98 (27.5%) subjects with MTBS, and 258 (72.5%) without MTBS, mainly of male gender (244/356, 68.5%) and with a mean age of 49 years (standard deviation, SD: 12). Demographics, lifestyle, and clinical characteristics of the study population are depicted in Table 1 according to the presence of metabolic syndrome (PLWH with MTBS and PLWH without MTBS). In the MTBS group, PLWH had a mean age of 53 years (SD: 10), were mainly of male gender (76.5%), and experienced AIDS events in almost 90% cases. PLWH without MTBS had a mean age of 47.6 (SD: 11.6) and were mainly of male gender (65.5%). Prevalence of previous AIDS events in this groups was 27.5%.

At the univariate analysis (Table 2), factors significantly associated with MTBS were age (53.1 vs. 47.6, *p* < 0.001), male gender (OR: 0.58, 95% CI: 0.3–1.1, *p* = 0.04), excessive alcohol intake (OR: 2.1, 95% CI: 1.3–3.5, *p* = 0.019), chronic pulmonary disease (OR: 2.56, 95% CI: 1.1–5.7, *p* = 0.01), neurological diseases (OR: 3.4, 95% CI: 1.6–7.1, *p* = 0.002), osteoporosis (OR: 3.42, 95% CI 1.8–6.4, *p* < 0.01), polypharmacy (OR: 14.3, 95% CI 4.4–59.2, *p* < 0.01), and AIDS events in the past medical history (OR: 23.1, 95% CI 11.1–52, *p* < 0.01). At the multivariable model, (Table 2), significant association was maintained only for alcohol consumption (OR: 3.1, 95% CI 1.4–6.6; *p* < 0.01), osteoporosis (OR: 3.1, 95% CI 1.8–7.3, *p* < 0.01), polypharmacy (OR: 7.1, 95% CI: 1.85–27.6; *p* < 0.01), and history of AIDS events (OR: 21, 95% CI 10.9–44.1, *p* < 0.01).

## 4. Discussion

We found that approximately one-third (27.5%) of PLWH in our cohort from southern Italy had MTBS. This result in the middle of the range of prevalence of MTBS in the general population, which is from 15% to 29% in Italy [22,23,24]. As for PLWH, in comparison with other cohorts from Mediterranean area, (i.e., Spain), where the prevalence was 11.4%, our prevalence of MTBS was higher [25], while it was lower than that recently described by a multicenter Italian cohort reporting a prevalence of MTBS of 29.3–35% in PLWH over 10 years [12]. According to the latter study, prevalence of MTBS in PLWH residing in Italy decreased from 2005 to 2015. However, since then, a new class of antiretroviral, the integrase inhibitors, which are strictly associated with weight gain, is available. Whether the advent of this new class influenced the prevalence of MTBS in PLWH across Italy remains to be investigated. 

Due to the residency of our patients in the Mediterranean area, we would have expected a far lower prevalence of MTBS, similar to that found in other Mediterranean cohorts (15–21%) [22,23]. A plausible explanation for this discrepancy could be the indirect effect of globalization that has also changed people’s eating habits (increased consumption of “junk food “and sweet/carbonated drinks) [26]. On the other hand, more likely, it could be due to social determinants recently identified as determinants of MTBS in the Italian Obesity Barometer Report 2019 [27]. Herein, it is demonstrated that 30% of Italians are overweighted/obese and that proportions of obese/overweighted people is greater in the south compared to the north of Italy [27]. This difference is due to sedentary lifestyle, lower level of education, and high caloric intake [27].

This risk factors are represented also in PLWH [28,29] and confirmed by our results. Moreover, in PLWH, some lifestyle behaviors increasing the risk of MTBS (such as alcohol abuse) are overrepresented, increasing the risk of metabolic disorders further [30,31,32]. In our cohort, people with excessive alcohol intake were 3.1-fold more likely to have MTBS when compared to those who did not report any alcohol consumption. Moreover, alcohol consumption is a part of the nutritional habit; hence, it is likely that these factors may influence each other. Therefore, educational interventions to avoid and control alcohol abuse should be promoted.

Another crucial tool to prevent MTBS is performing regular exercise, which also prevents other comorbidities such as bone disorders, specifically osteoporosis, and could contribute to keeping ageing people fit.

Polypharmacy was significantly associated with MTBS in our cohort, and this could be easily explained by the fact that polypharmacy is a proxy of comorbidities. Moreover, the use of specific medications (protease inhibitors, antidepressants agents, corticosteroids, oral contraceptives) may increase the risk of the development of the metabolic syndrome by either promoting weight gain or altering lipid or glucose metabolism [33,34]. Healthcare providers should promptly recognize, systematically review, and assess the risk associated with some medications more than others and appropriately change/switch off medications contributing to the burden of metabolic disease. Moreover, careful attention to the drug choices should be paid in patients who are overweight or have other risk factors for diabetes or cardiovascular disease.

Our data showed a significant association between MTBS and previous AIDS events. Furthermore, in our analysis, a trend to significance was found for CD4/CD8 ratio: PLWH with a low CD4/CD8 ratio (<1) were more likely to have MTBS (*p* = 0.05). A low CD4/CD8 ratio has been linked to ageing and acts both as a predictor of mortality in the general population and a biomarker of inflammation in PLWH [35]. It would therefore seem that both inflammation and immunosuppression play a role in metabolic diseases.

It should be noted that our analysis shows that (even if not statistically) the most educated people tend to have half the risk of developing MTBS. A possible explanation is that highly educated people take much more care regarding quality of food and lifestyles; by contrast, people with lower levels of education may eat larger amounts of unhealthy, calorically dense food than those with a higher education level [36]. This result is also in line with data from the general Italian population previously mentioned.

This study is somewhat limited by its cross-sectional nature, by the lack of a control group, by the low number of participants, and by the possible bias connected with a retrospective collection of data from clinical health records (missing information such as underreporting of comedications and comorbidities, etc.). Furthermore, categorizations of some variables in a dichotomous way could have had an impact on our results [37]. Moreover, it is well-recognized that there are prominent sex differences in MTBS [38,39]. Given the prominent sex differences in the pathogenesis of metabolic syndrome, it is possible that the risk factors associated with MTBS may also be altered by different gender distribution, and this can also be seen as a limitation of our study in terms of generalizability of results.

Our study suggests that prevalence of MTBS in our cohort is high (27.5%); therefore, it is important to both identify risk factors and implement educational/multidimensional interventions to prevent MTBS in PLWH, especially for those with particular risk factors (previous AIDS events or polypharmacy). Moreover, some behaviors, such as alcohol consumption, should be routinely investigated in clinical practice, and campaigns should be implemented to promote a change in the lifestyle of patients by promoting healthy diets, weight loss, and physical activity. Lastly, since available data are still debated, more recent and updated data are necessary to establish the actual prevalence of MTBS in PWLH.

## Figures and Tables

**Table 1:** Baseline characteristics by presence of metabolic syndrome.

Variable	No. PLWH with MTBS (%) 98 (100)	No. PLWH without MTBS (%) 258 (100)	*p*
Age, mean (SD)	53.1 (10.3)	47.6 (11.6)	<0.001
Male gender	75 (76.5)	169 (65.5)	0.04
Country (Italy)	93 (94.9)	217 (84.1)	0.006
Highest level of education	12 (12.1)	54 (20.9)	0.05
Living alone	54 (55.1)	153 (59.3)	0.47
Being retired	15 (15.3)	22 (8.5)	0.05
Being smoker	57 (58.2)	130 (50.4)	0.18
Doing regular exercise	25 (25.5)	89 (34.5)	0.104
Excessive alcohol intake	51 (52)	88 (34.1)	0.019
Chronic kidney disease	10 (10.2)	20 (7.7)	0.45
Cirrhosis	3 (3.1)	5 (1.9)	0.52
COPD	15 (15.3)	17 (6.6)	0.01
Malignancies	3 (3.1)	5 (1.9)	0.52
Psychiatric disorders	24 (24.5)	65 (25.2)	0.89
Neurological disorders	21 (21.4)	19 (7.4)	0.002
Osteoporosis	28 (28.6)	27 (10.5)	<0.01
Thyroid diseases	4 (4.1)	11 (4.3)	0.93
HBV coinfection	7 (7.1)	21 (8.1)	0.75
HCV coinfection	27 (27.5)	59 (22.9)	0.35
HBV/HCV coinfection	4 (4.1)	5 (1.9)	0.249
Polypharmacy	18 (18.4)	4 (1.5)	<0.01
CD4/CD8 ratio > 1	20 (20.1)	79 (30.6)	0.05
Previous AIDS events	88 (89.9)	71 (27.5)	<0.01
HIV RNA > 50 copies/mL	5 (5.1)	13 (5.1)	0.98
Years with HIV, mean (SD)	15.9 (0.6)	14.2 (0.6)	0.9
Last CD4 T cell count, mean (SD)	669 (21)	705 (37)	0.8
CD4 T cell count nadir, mean (SD)	310 (15)	277 (23)	0.13
cART *			
2NRTI + INI	47 (47.9)	118 (45.7)	0.7
2NRTI + NNRTI	13 (13.2)	53 (20.5)	0.2
2NRTI + PI	18 (18.4)	48 (19.8)	0.9
INI + PI	7 (7.3)	22 (8.5)	0.7
Dual	0 (0)	5 (1.9)	0.2

SD, standard deviation; PLWH, people living with HIV; MTBS, metabolic syndrome; COPD, chronic obstructive pulmonary disease; cART, combination antiretroviral therapy; NRTI, nucleos(t)ide reverse transcriptase inhibitors; NNRTI, non-nucleos(t)ide reverse transcriptase inhibitors; INI, integrase inhibitors; PI, protease inhibitors. *, 13 subjects in the MTBS group and 12 in the group without MTBS were receiving cART not present in the listed combinations.

**Table 2:** Univariate and multivariate analyses of risk factors associated with metabolic syndrome in PLWH.

Variable	No. PLWH with MTBS (%) 98 (100)	No. PLWH without MTBS (%) 258 (100)	Univariable Analysis		Multivariable Analysis	
			Odds Ratio (95% CI)	*p*	Odds Ratio (95% CI)	*p*
Age, mean (SD)	53.1 (10.3)	47.6 (11.6)	-	<0.001		
Male gender	75 (76.5)	169 (65.5)	0.58 (0.3–1.1)	0.04		
Country (Italy)	93 (94.9)	217 (84.1)	3.5 (1.32–11.7)	0.006		
Highest level of education	12 (12.1)	54 (20.9)	0.52 (0.24–1.1)	0.05		
Living alone	54 (55.1)	153 (59.3)	0.84 (0.51–1.4)	0.47		
Being retired	15 (15.3)	22 (8.5)	1.9 (0.88–4.1)	0.05		
Being smoker	57 (58.2)	130 (50.4)	1.36 (0.8–2.25)	0.18		
Doing regular exercise	25 (25.5)	89 (34.5)	0.66 (0.36–1.1)	0.104		
Excessive alcohol intake	51 (52)	88 (34.1)	2.1 (1.3–3.5)	0.019	3.1 (1.4–6.6)	<0.01
Chronic kidney disease	10 (10.2)	20 (7.7)	1.35 (0.54–3.2)	0.45		
Cirrhosis	3 (3.1)	5 (1.9)	1.59 (0.24–8.4)	0.52		
COPD	15 (15.3)	17 (6.6)	2.56 (1.1–5.7)	0.01		
Malignancies	3 (3.1)	5 (1.9)	1.59 (0.24–8.4)	0.52		
Psychiatric disorders	24 (24.5)	65 (25.2)	0.96 (0.53–1.7)	0.89		
Neurological disorders	21 (21.4)	19 (7.4)	3.4 (1.6–7.1)	0.002		
Osteoporosis	28 (28.6)	27 (10.5)	3.42 (1.8–6.4)	<0.01	3.6 (1.8–7.3)	<0.01
Thyroid diseases	4 (4.1)	11 (4.3)	0.95 (0.21–3.3)	0.93		
HBV coinfection	7 (7.1)	21 (8.1)	0.86 (0.3–2.1)	0.75		
HCV coinfection	27 (27.5)	59 (22.9)	1.28 (0.7–2.24)	0.35		
HBV/HCV coinfection	4 (4.1)	5 (1.9)	2.1 (0.41–10.2)	0.249		
Polypharmacy	18 (18.4)	4 (1.5)	14.3 (4.4–59.2)	<0.01	7.1 (1.85–27.6)	<0.01
CD4/CD8 ratio > 1	20 (20.1)	79 (30.6)	0.58 (0.31–1.1)	0.05		
Previous AIDS events	88 (89.9)	71 (27.5)	23.1 (11.1–52)	<0.01	21 (10.9–44.1)	<0.01
HIV RNA > 50 copies/mL	5 (5.1)	13 (5.1)	1.01 (0.27–3.13)	0.98		
Years with HIV, mean (SD)	15.9 (0.6)	14.2 (0.6)	-	0.9		
Last CD4 T cell count, mean (SD)	669 (21)	705 (37)	-	0.8		
CD4 T cell count nadir, mean (SD)	310 (15)	277 (23)	-	0.13		
cART *						
2NRTI + INI	47 (47.9)	118 (45.7)	1.1 (0.66–1.78)	0.7		
2NRTI + NNRTI	13 (13.2)	53 (20.5)	0.5 (0.3–1.2)	0.2		
2NRTI + PI	18 (18.4)	48 (19.8)	0.9 (0.5–1.8)	0.9		
INI + PI	7 (7.3)	22 (8.5)	0.8 (0.3–2.1)	0.7		
Dual	0 (0)	5 (1.9)	0 (0–2)	0.2		

SD, standard deviation; PLWH, people living with HIV; MTBS, metabolic syndrome; COPD, chronic obstructive pulmonary disease; cART, combination antiretroviral therapy; NRTI, nucleos(t)ide reverse transcriptase inhibitors; NNRTI, non-nucleos(t)ide reverse transcriptase inhibitors; INI, integrase inhibitors; PI, protease inhibitors. *, 13 subjects in the MTBS group and 12 in the group without MTBS were receiving cART not present in the listed combinations.

## Data Availability

All data of interest are herein reported.
